# Supplementary material for: Multiomics reveals microbial metabolites as key actors in intestinal fibrosis in Crohn’s disease
Source: EMBO Mol Med. 2024 Sep 13;16(10):11. doi: 10.1038/s44321-024-00129-8 (PMC11473649; doi:10.1038/s44321-024-00129-8)
Supplement: Supplementary file 1 — Appendix [file 44321_2024_129_MOESM1_ESM.pdf]

Table of content of the Appendix Tables

Page

Appendix Table S1……………………………………………………………2

Appendix Table S2……………………………………………………………3

Appendix Table S3……………………………………………………………4

Appendix Table S4……………………………………………………………6

Appendix Table S5……………………………………………………………6

Appendix Table S1. Exact *P*-values presented in Figure 1.

Figure 1A

Alpha diversity	BF1_HC_Pvalue	BF2_HC_Pvalue	BF1_BF2_Pvalue
Chao1	3.78651E-08	1.98703E-12	0.082054337
Simpson	1.48166E-05	4.14443E-10	0.021716701
Shannon	3.66153E-07	6.20027E-12	0.036526799
Pielou_e	5.62915E-06	1.66869E-10	0.027993185

Figure 1C

bacterial genera	BF2_HC_Pvalue
Romboutsia	0.01757
Lachnospiraceae_NK4A136_group	0.000787
Ruminococcus_2	0.00182
Ruminococcaceae_UCG_004	0.009811
Muribaculaceae	0.031661
Anaerotruncus	0.038168
Mogibacterium	0.041647

Figure 1D

fecal metabolite	BF2_HC_Pvalue
Xylose	0.038258

Figure 1E

blood metabolites	BF2_HC_Pvalue
Glutamine	0.03279
Serine	0.053104
N-Acetylserine	0.015465
3-Hydroxylisovalerylcarnitine	0.034485
Rhamnose	0.030076
LPE (16:0)	0.023166
LPC (16:1)	0.044144
LPC (18:1)	0.04324
LPC (20:0)	0.017898
LPC (22:6)	0.05422
TG (52:6)	0.068839
TG (53:4)	0.039603

Appendix Table S2. Exact *P*-values presented in Figure 5.

Figure 5B

	Week2		
	C0_C1_Pvalue	C1_Tx_Pvalue	C0_Tx_Pvalue
SUV-max	0.022	0.01	0.004
	Week3		
	C0_C1_Pvalue	C1_Tx_Pvalue	C0_Tx_Pvalue
SUV-max	<0.001	0.007	0.001

Figure 5C

	Week2		
	C0_C1_Pvalue	C1_Tx_Pvalue	C0_Tx_Pvalue
Normalized MTR	0.155	0.04	0.01
	Week3		
	C0_C1_Pvalue	C1_Tx_Pvalue	C0_Tx_Pvalue
Normalized MTR	0.013	<0.001	0.003

Figure 5D

	Week2		
	C0_C1_Pvalue	C1_Tx_Pvalue	C0_Tx_Pvalue
Fibrotic score		0.583	
	Week3		
	C0_C1_Pvalue	C1_Tx_Pvalue	C0_Tx_Pvalue
Fibrotic score		0.481	

Appendix Table S3. Exact *P*-values presented in Figure 6.

Figure 6A

	Group1_Group2_Pvalue	Group1_Group3_Pvalue	Group1_Group4_Pvalue	Group2_Group3_Pvalue	Group3_Group4_Pvalue	Group2_Group4_Pvalue
COL1A1	<0.001	<0.001	<0.001	0.009	<0.001	<0.001

Figure 6B

	Group1_Group2_Pvalue	Group1_Group3_Pvalue	Group1_Group4_Pvalue	Group2_Group3_Pvalue	Group3_Group4_Pvalue	Group2_Group4_Pvalue
FN	<0.001	<0.001	<0.001	0.033	0.045	<0.001

Figure 6C

	Group1_Group2_Pvalue	Group1_Group3_Pvalue	Group1_Group4_Pvalue	Group2_Group3_Pvalue	Group3_Group4_Pvalue	Group2_Group4_Pvalue
ACTA2	<0.001	<0.001	<0.001	0.618	0.041	0.003

Figure 6E

	Group1_Group2_Pvalue	Group1_Group3_Pvalue	Group1_Group4_Pvalue	Group2_Group3_Pvalue	Group3_Group4_Pvalue	Group2_Group4_Pvalue
COL1A1	<0.001	<0.001	<0.001	0.031	0.003	<0.001

Figure 6F

	Group1_Group2_Pvalue	Group1_Group3_Pvalue	Group1_Group4_Pvalue	Group2_Group3_Pvalue	Group3_Group4_Pvalue	Group2_Group4_Pvalue
FN	<0.001	<0.001	<0.001	0.043	0.112	<0.001

Note: Group1: *L*-aspartic acid (-) +TGF-β1(-); Group2: *L*-aspartic acid (-) +TGF-β1(+); Group3: *L*-aspartic acid(6.5μM) +TGF-β1(+); Group4: *L*-aspartic acid(13μM) +TGF-β1(+).

Appendix Table S4. Exact *P*-values presented in Figure EV1.

Figure EV1 C

Group	Criteria1_Criteria2_Pvalue
BF1	0.015
BF2	0.005

Figure EV1 D

Group	Criteria1_Criteria2_Pvalue
BF1	0.001
BF2	0.000001

Appendix Table S5. Exact *P*-values presented in Figure EV3.

Figure EV3

HC_BI1_Pvalue	BI1_BI2_Pvalue	HC_BI2_Pvalue
0.001	0.708	0.001
