# Supplementary material for: Multiomics reveals microbial metabolites as key actors in intestinal fibrosis in Crohn’s disease
Source: EMBO Mol Med. 2024 Sep 13;16(10):11. doi: 10.1038/s44321-024-00129-8 (PMC11473649; doi:10.1038/s44321-024-00129-8)
Supplement: Supplementary file 2 — Table EV1 [file 44321_2024_129_MOESM2_ESM.docx]

**Table EV1. Clinical characteristics of healthy controls and patients with CD**

| Characteristics | Healthy controls | Derivation cohort | Test cohort | *P* ^b^ |
| --- | --- | --- | --- | --- |
|  | (n=28) | (n=214) | (n=64) |  |
| **Gender, n (%)** |  |  |  | 0.904 |
| Male | 23 (82.14) | 172 (80.37) | 51 (79.69) |  |
| Female | 5 (17.86) | 42 (19.63) | 13 (20.31) |  |
| **Age, mean ± SD/median [IQR]** | 29.86±6.08 | 30.00 [24.00,36.00] | 28.00 [22.00,35.00] | 0.145 |
| **Disease course, month, median [IQR]** |  | 48.00 [12.50, 96.00] | 36.00 [12.00,85.50] | 0.317 |
| **Drug use ^a^, n (%)** |  |  |  | 0.031 |
| Biologics |  | 60 (28.04) | 25 (39.06) |  |
| Corticosteroids |  | 10 (4.67) | 2 (3.13) |  |
| Immunomodulator |  | 62 (28.97) | 9 (14.06) |  |
| 5-Aminosalicylic acid |  | 35 (16.36) | 17 (26.56) |  |
| **Surgery, n (%)** |  |  |  | 0.758 |
| Intestinal surgery |  | 54 (25.23) | 10 (15.63) |  |
| Perianal surgery |  | 32 (14.95) | 7 (10.94) |  |
| **Montreal classification, n (%)** |  |  |  |  |
| *Age* |  |  |  | 0.649 |
| A1 [≤16] |  | 4 (1.87) | 0 (0.00) |  |
| A2 [17-40] |  | 188 (87.85) | 59 (92.19) |  |
| A3 [>40] |  | 22 (10.28) | 5 (7.81) |  |
| *Disease location* |  |  |  | 0.283 |
| L1 [terminal ileum] |  | 23 (10.75) | 10 (15.62) |  |
| L2 [colon] |  | 8 (3.73) | 3 (4.69) |  |
| L3 [ileocolon] |  | 174 (81.31) | 51 (79.69) |  |
| L4 [upper gastrointestinal] |  | 9 (4.21) | 0 (0.00) |  |
| *Disease behavior* |  |  |  | 0.146 |
| B1 [inflammatory] |  | 81 (37.85) | 28 (43.75) |  |
| B2 [stricturing] |  | 66 (30.84) | 24 (37.50) |  |
| B3 [penetrating] |  | 67 (31.31) | 12 (18.75) |  |
| **Perianal diseases, n (%)** |  |  |  | 0.663 |
| Fistula |  | 104 (48.60) | 31 (48.44) |  |
| Abscess |  | 25 (11.68) | 6 (9.38) |  |
| **Smoking, n (%)** | 4 (14.29) | 20 (9.35) | 3 (4.69) | 0.235 |
| **Drinking, n (%)** | 12 (42.86) | 15 (7.01) | 2 (3.13) | 0.401 |
| **BMI, kg/m^2^, mean ± SD/median [IQR]** | 23.31±1.85 | 19.07 [17.59, 21.30] | 19.04 [17.35,20.68] | 0.418 |

^a^ Medicine use within 3 months before inclusion.

^b^ Comparison between derivation cohort and test cohort.

CD, Crohn’s disease; BMI, Body mass index; SD, Standard deviation; IQR, interquartile range
