# Supplementary material for: Multiomics reveals microbial metabolites as key actors in intestinal fibrosis in Crohn’s disease
Source: EMBO Mol Med. 2024 Sep 13;16(10):11. doi: 10.1038/s44321-024-00129-8 (PMC11473649; doi:10.1038/s44321-024-00129-8)
Supplement: Supplementary file 11 — Table EV10 [file 44321_2024_129_MOESM11_ESM.docx]

**Table EV10. Assessment of magnetic resonance enterography features in intestinal strictures**

| **Semi-quantitative evaluation** | **Score 0** | **Score 1** | **Score 2** | **Median [IQR] of scores** |
| --- | --- | --- | --- | --- |
| **Bowel stricture**  (1) Luminal narrowing: luminal diameter reduction by at least 50%, measured relative to an appropriately distended normal bowel.  (2) Upstream dilation: bowel diameter is 50% greater than the diameter in an appropriately distended normal lumen. | 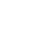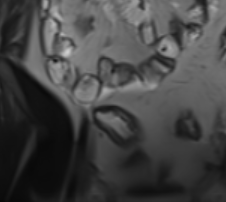  No luminal narrowing, no upstream dilation | 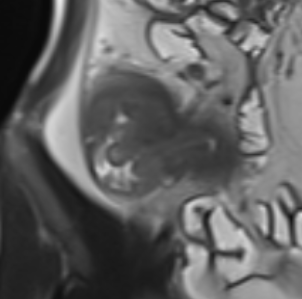  Luminal narrowing without upstream dilation | 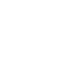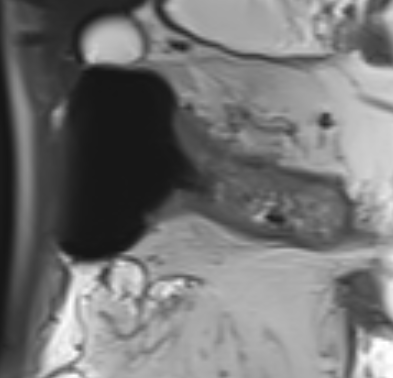  8.15 cm  Luminal narrowing with upstream dilation | 1.00  [0.00,2.00] |
| **Penetrating diseases**  (1) Ulceration: small focal breaks in the mucosal surface of bowel wall with focal extension of air or enteric contrast into the inflamed bowel wall.  (2) Fistula: appears as an extra-enteric tract, with or without internal air or fluid or multiple tracts often forming an asterisk-shaped or ‘clover-leaf’ appearance, or ‘star sign’; affected loops are often angulated or tethered.  (3) Abscess: mesenteric or peritoneal fluid collection with rim enhancement and/or internal air. | 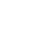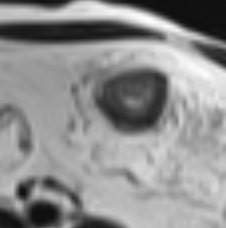  Absent of penetrating diseases | 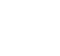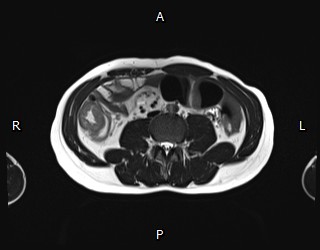  Deep ulceration | 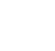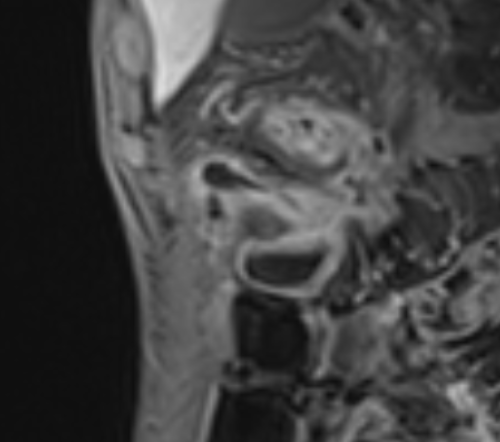  Fistula/abscess | 1.00  [1.00,1.00] |
| **Perienteric effusion**  Increased signal on fat-saturated T2-weighted imaging in mesenteric fat adjacent to abnormal bowel loops | 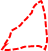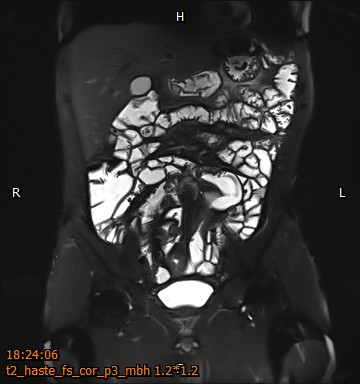  Absent of perienteric effusion | 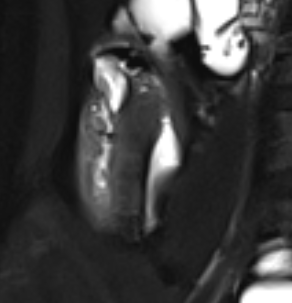  Effusion without fluid | 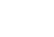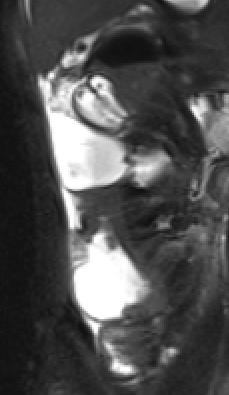  Effusion with fluid (arrow) | 0.00  [0.00,1.00] |
| **Mural enhancement degree in arterial phase** | 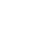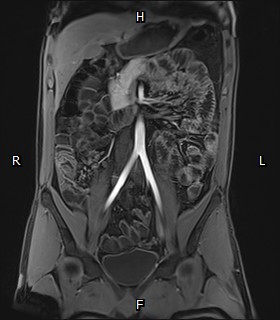  Similar signal intensity as normal bowel | 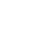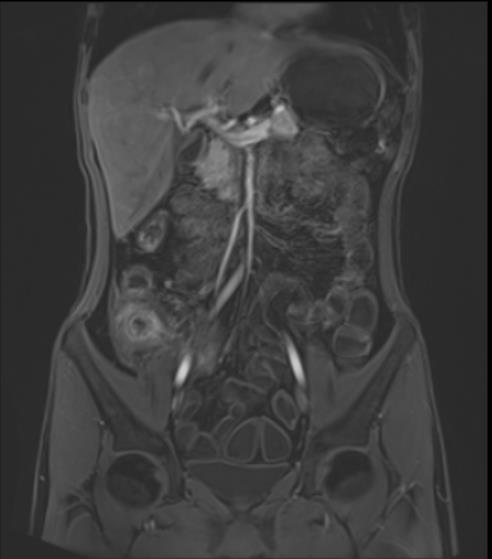  Slightly less than the signal intensity of artery | 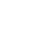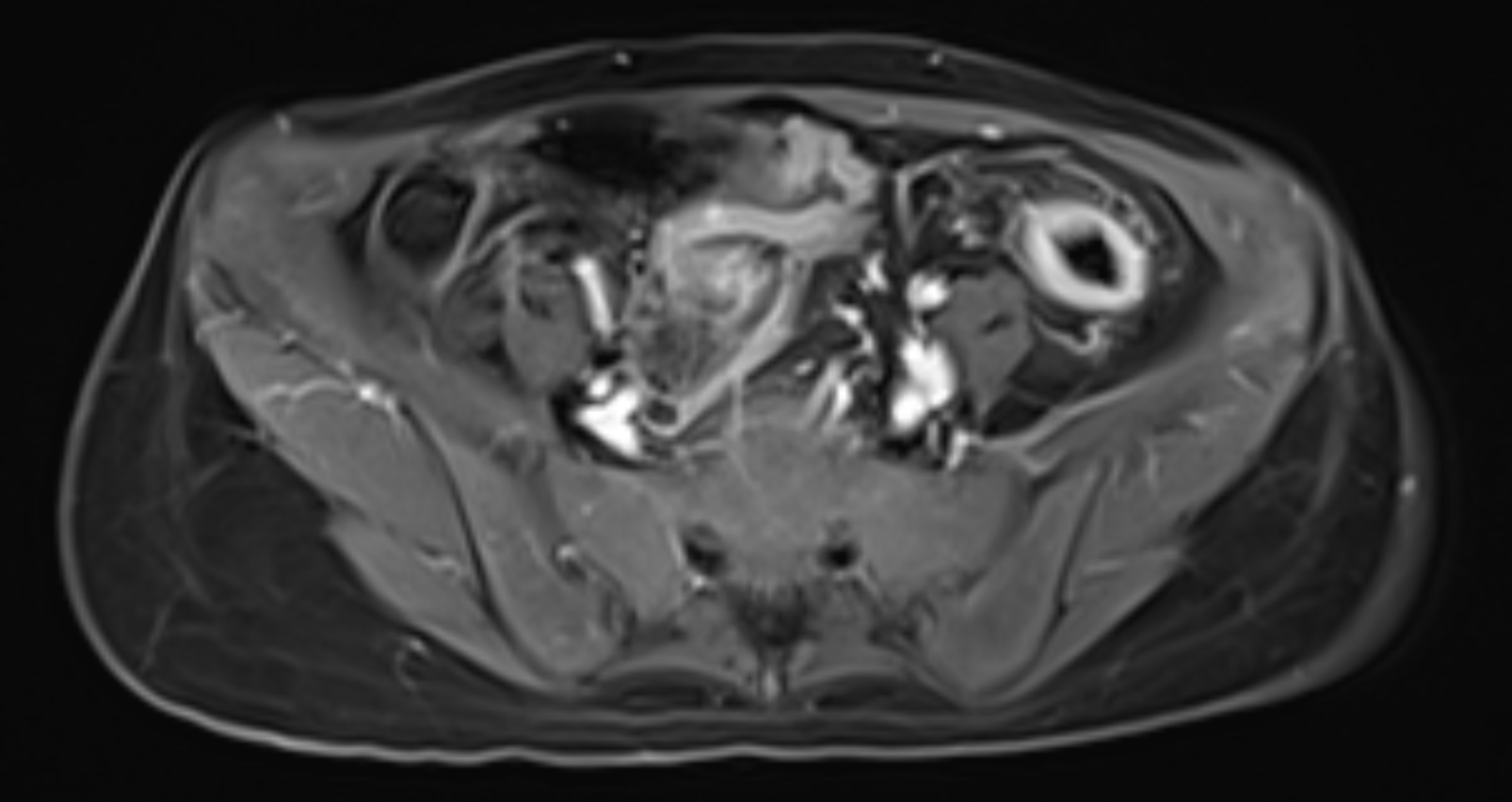  Equal to the signal intensity of artery | 1.00  [1.00,2.00] |
| **Perianal diseases** | 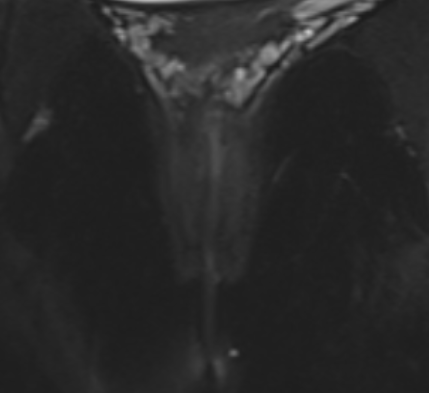  Absent of perianal disease | 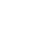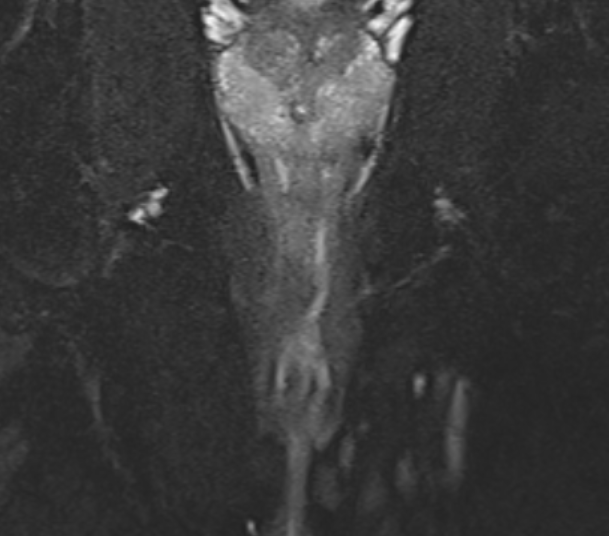  Perianal fistula | 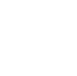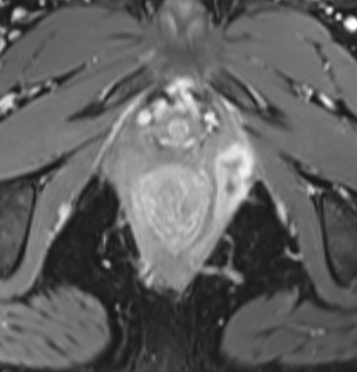  Perianal abscess | 1.00  [0.00,1.00] |
| **Comb sign**  Engorged vasa recta that supplying an inflamed bowel loop | 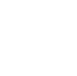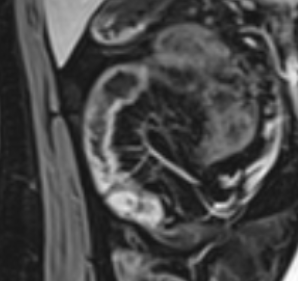  Absent of comb sign | 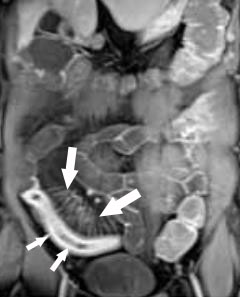  Present of comb sign |  | 0.00  [0.00,1.00] |
| **Mural edema**  Hyperintense signal on fat-saturated T2-weighted image | 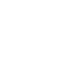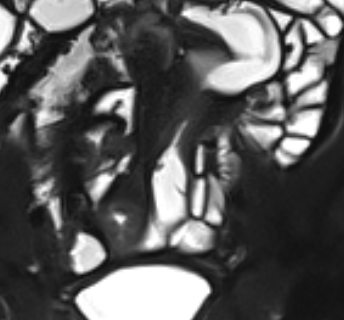  Absent of intramural edema | 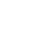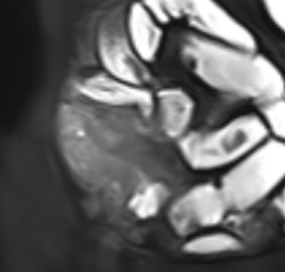  Present of intramural edema |  | 1.00  [0.00,1.00] |
| **Mural enhancement pattern** | 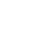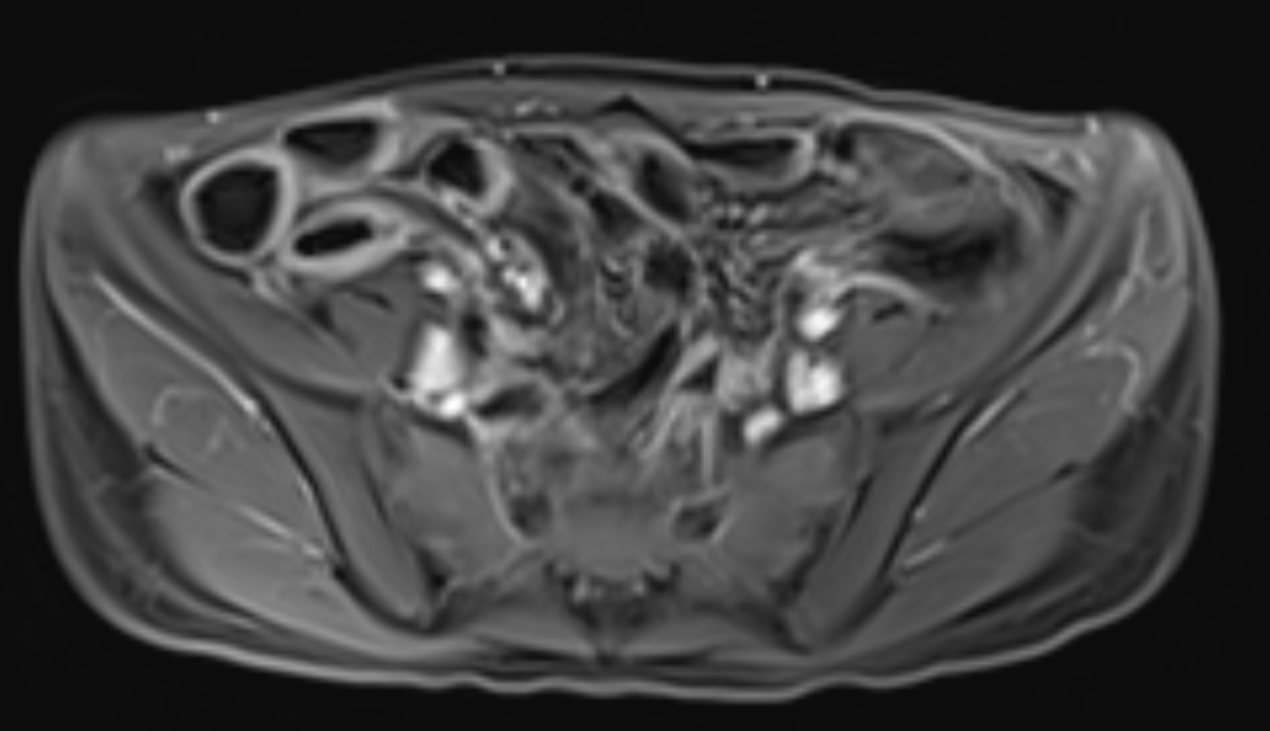  Homogeneous | 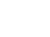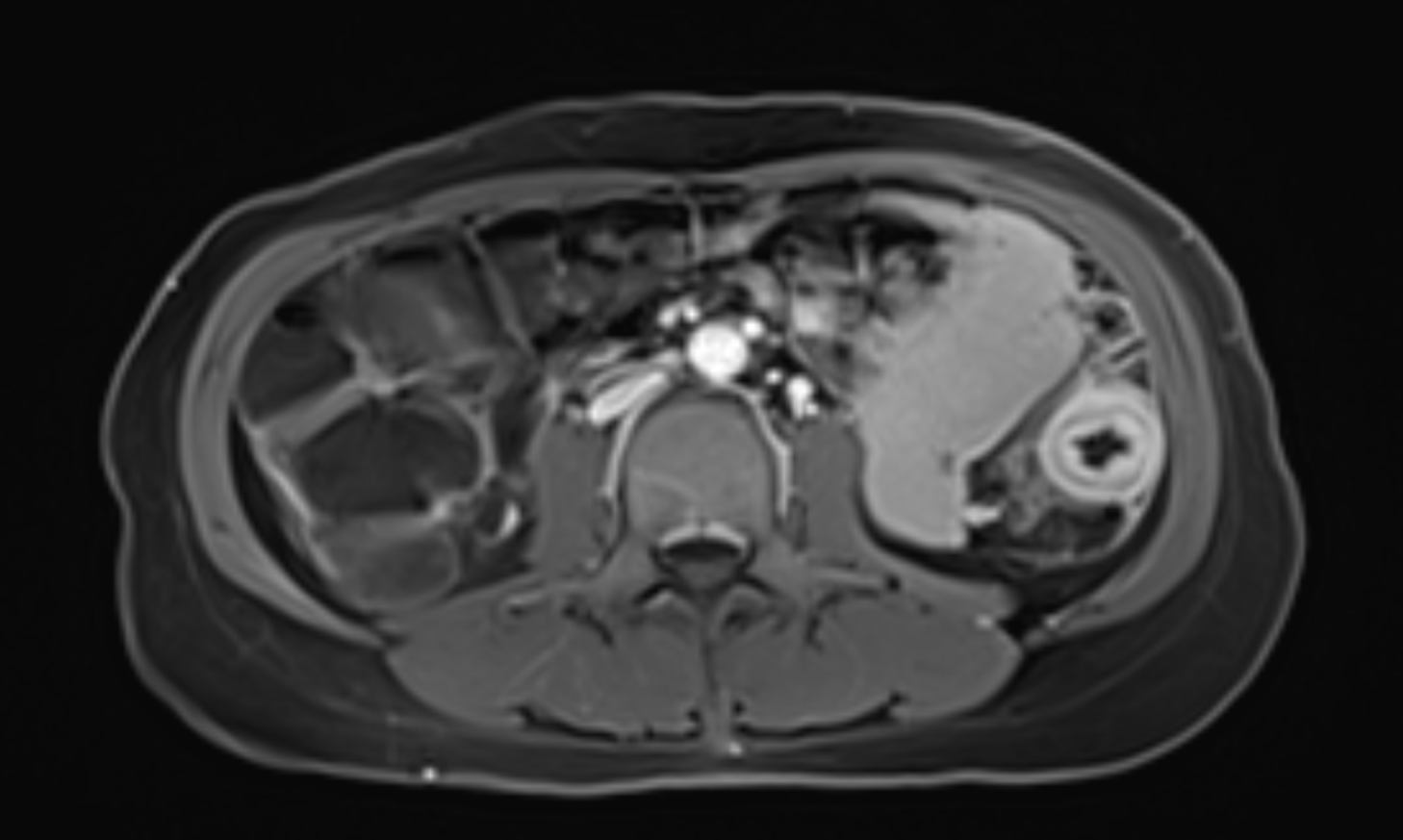  Stratified |  | 0.00  [0.00,0.00] |
| **Length of diseased bowel** | 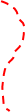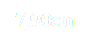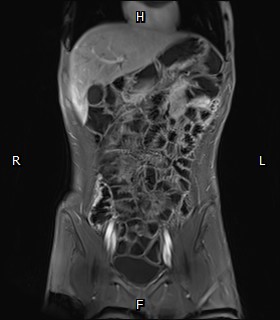  Length≤15 cm | 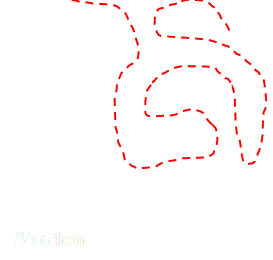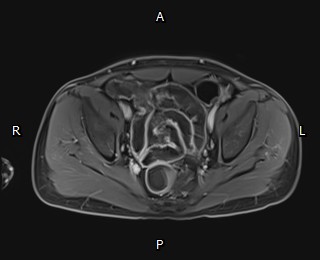  Length>15 cm |  | 0.00  [0.00,0.00] |
| **Adenopathy** | 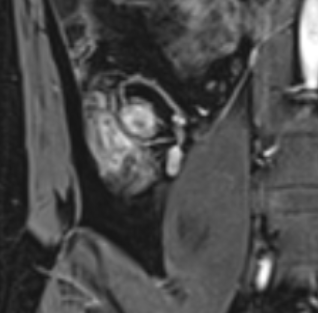  6 mm  Short axis of lymph node≤1 cm | 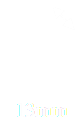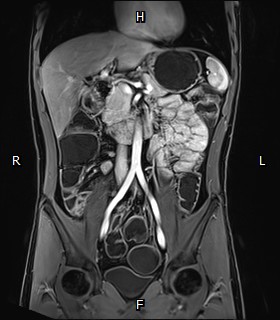  Short axis of lymph node>1 cm |  | 0.00  [0.00,0.00] |
| **Quantitative evaluation** | | | | **Mean ± SD** |
| **Wall thickness** | 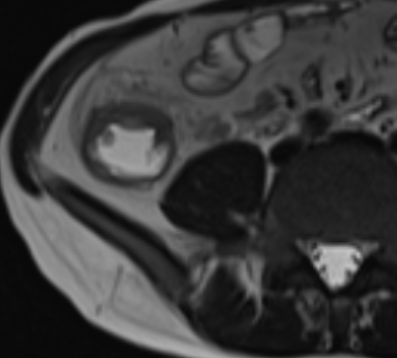  11 mm  Measured the largest bowel wall thickness on the thickest area on the short axis of inflamed intestine | | | (6.61±3.27) mm |
| **Mural apparent diffusion coefficient** | 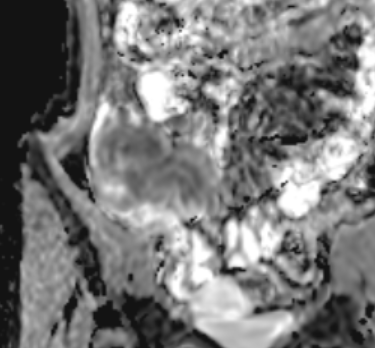  ADC=0.858×10^-3^ mm^2^/s  Measured on the most severe bowel segment | | | (1.17±0.31) ×10^-3^ mm^2^/s |

IQR, interquartile range; SD, Standard deviation
