# Supplementary material for: Multiomics reveals microbial metabolites as key actors in intestinal fibrosis in Crohn’s disease
Source: EMBO Mol Med. 2024 Sep 13;16(10):11. doi: 10.1038/s44321-024-00129-8 (PMC11473649; doi:10.1038/s44321-024-00129-8)
Supplement: Supplementary file 12 — Computer Code EV1 [file 44321_2024_129_MOESM12_ESM.zip › Computer Code EV1/README.docx]

**Supplementary data. Diagnostic classifier**

A classifier (which can be executed in Python after downloading the attachment in Supplementary data) for distinguishing the severity of intestinal fibrosis in patients with CD. The classifier was developed based on the combined random forest model with the 11 predictors (three genera [*Ruminococcaceae_UCG_003*, *Solobacterium*, *Erysipelatoclostridium*], two fecal metabolites [linoleylcarnitine and glycoursodeoxycholic acid] and six blood metabolites [citramalic acid, PE.36:2, PE.36:1, PE.36:0, PE.34:2, DAG.36:5]). The prediction of intestinal fibrosis can be automatically output based on the input of data for each patient regarding these 11 parameters.
