# Supplementary figures and images for: Multiomics reveals microbial metabolites as key actors in intestinal fibrosis in Crohn’s disease
Source: EMBO Mol Med. 2024 Sep 13;16(10):11. doi: 10.1038/s44321-024-00129-8 (PMC11473649; doi:10.1038/s44321-024-00129-8)

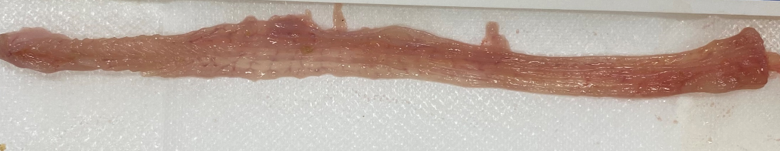

Supplement: Supplementary file 30 — Source data Fig. 5 [file 44321_2024_129_MOESM30_ESM.zip › Source data_Figure5/5A/C0 Week2.tif]

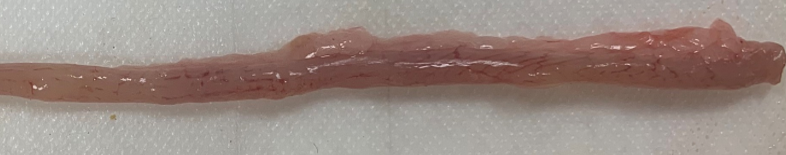

Supplement: Supplementary file 30 — Source data Fig. 5 [file 44321_2024_129_MOESM30_ESM.zip › Source data_Figure5/5A/C0 Week3.tif]

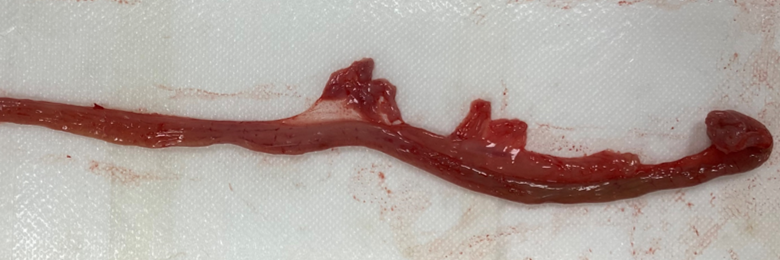

Supplement: Supplementary file 30 — Source data Fig. 5 [file 44321_2024_129_MOESM30_ESM.zip › Source data_Figure5/5A/C1 Week2.tif]

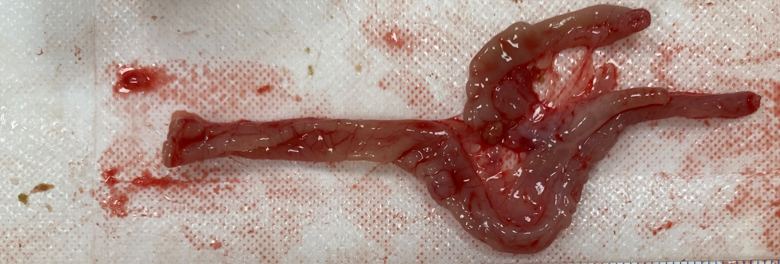

Supplement: Supplementary file 30 — Source data Fig. 5 [file 44321_2024_129_MOESM30_ESM.zip › Source data_Figure5/5A/C1 Week3.tif]

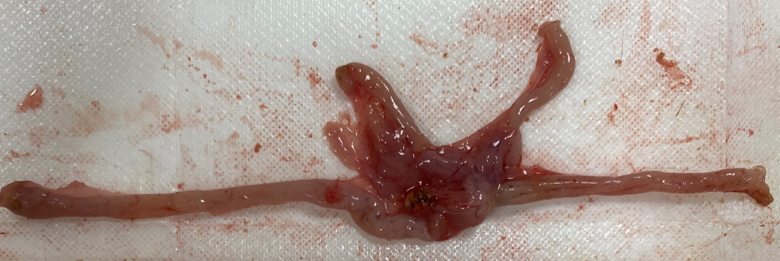

Supplement: Supplementary file 30 — Source data Fig. 5 [file 44321_2024_129_MOESM30_ESM.zip › Source data_Figure5/5A/Tx Week2.tif]

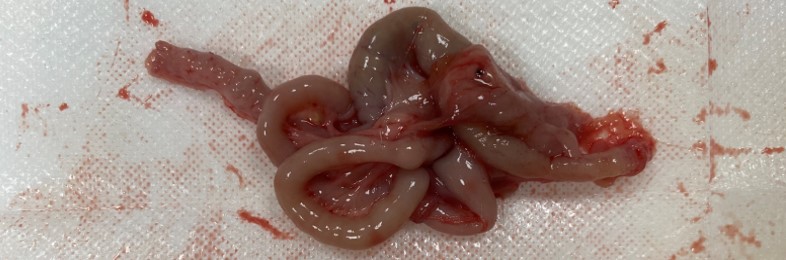

Supplement: Supplementary file 30 — Source data Fig. 5 [file 44321_2024_129_MOESM30_ESM.zip › Source data_Figure5/5A/Tx Week3.jpg]

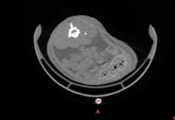

Supplement: Supplementary file 30 — Source data Fig. 5 [file 44321_2024_129_MOESM30_ESM.zip › Source data_Figure5/5B/5B Images/C0 Week2 CT_Axis.tif]

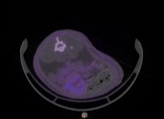

Supplement: Supplementary file 30 — Source data Fig. 5 [file 44321_2024_129_MOESM30_ESM.zip › Source data_Figure5/5B/5B Images/C0 Week2 PET_Axis.tif]

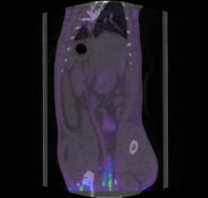

Supplement: Supplementary file 30 — Source data Fig. 5 [file 44321_2024_129_MOESM30_ESM.zip › Source data_Figure5/5B/5B Images/C0 Week2.tif]

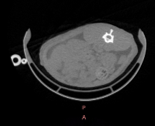

Supplement: Supplementary file 30 — Source data Fig. 5 [file 44321_2024_129_MOESM30_ESM.zip › Source data_Figure5/5B/5B Images/C0 Week3 CT_Axis.tif]

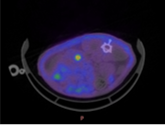

Supplement: Supplementary file 30 — Source data Fig. 5 [file 44321_2024_129_MOESM30_ESM.zip › Source data_Figure5/5B/5B Images/C0 Week3 PET_Axis.tif]

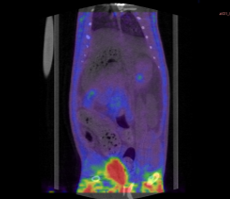

Supplement: Supplementary file 30 — Source data Fig. 5 [file 44321_2024_129_MOESM30_ESM.zip › Source data_Figure5/5B/5B Images/C0 Week3.tif]

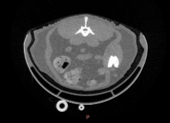

Supplement: Supplementary file 30 — Source data Fig. 5 [file 44321_2024_129_MOESM30_ESM.zip › Source data_Figure5/5B/5B Images/C1 Week2 CT_Axis.tif]

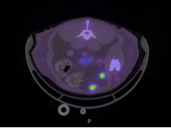

Supplement: Supplementary file 30 — Source data Fig. 5 [file 44321_2024_129_MOESM30_ESM.zip › Source data_Figure5/5B/5B Images/C1 Week2 PET_Axis.tif]

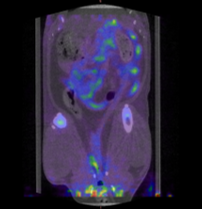

Supplement: Supplementary file 30 — Source data Fig. 5 [file 44321_2024_129_MOESM30_ESM.zip › Source data_Figure5/5B/5B Images/C1 Week2.tif]

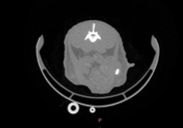

Supplement: Supplementary file 30 — Source data Fig. 5 [file 44321_2024_129_MOESM30_ESM.zip › Source data_Figure5/5B/5B Images/C1 Week3 CT_Axis.tif]

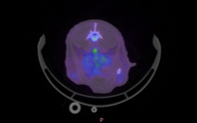

Supplement: Supplementary file 30 — Source data Fig. 5 [file 44321_2024_129_MOESM30_ESM.zip › Source data_Figure5/5B/5B Images/C1 Week3 PET_Axis.tif]

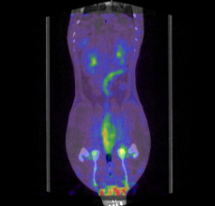

Supplement: Supplementary file 30 — Source data Fig. 5 [file 44321_2024_129_MOESM30_ESM.zip › Source data_Figure5/5B/5B Images/C1 Week3.tif]

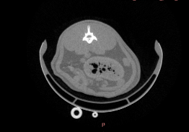

Supplement: Supplementary file 30 — Source data Fig. 5 [file 44321_2024_129_MOESM30_ESM.zip › Source data_Figure5/5B/5B Images/Tx Week2 CT_Axis.tif]

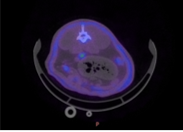

Supplement: Supplementary file 30 — Source data Fig. 5 [file 44321_2024_129_MOESM30_ESM.zip › Source data_Figure5/5B/5B Images/Tx Week2 PET_Axis.tif]

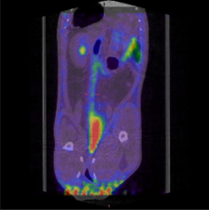

Supplement: Supplementary file 30 — Source data Fig. 5 [file 44321_2024_129_MOESM30_ESM.zip › Source data_Figure5/5B/5B Images/Tx Week2.tif]

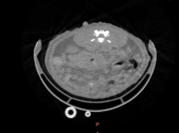

Supplement: Supplementary file 30 — Source data Fig. 5 [file 44321_2024_129_MOESM30_ESM.zip › Source data_Figure5/5B/5B Images/Tx Week3 CT_Axis.tif]

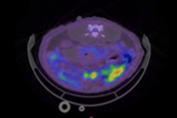

Supplement: Supplementary file 30 — Source data Fig. 5 [file 44321_2024_129_MOESM30_ESM.zip › Source data_Figure5/5B/5B Images/Tx Week3 PET_Axis.tif]

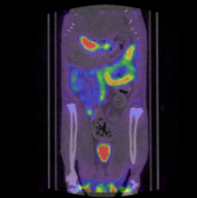

Supplement: Supplementary file 30 — Source data Fig. 5 [file 44321_2024_129_MOESM30_ESM.zip › Source data_Figure5/5B/5B Images/Tx Week3.tif]

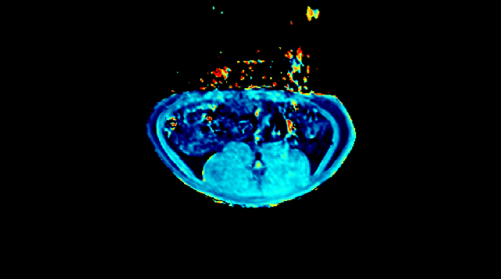

Supplement: Supplementary file 30 — Source data Fig. 5 [file 44321_2024_129_MOESM30_ESM.zip › Source data_Figure5/5C/5C Images/C0 Week2.tif]

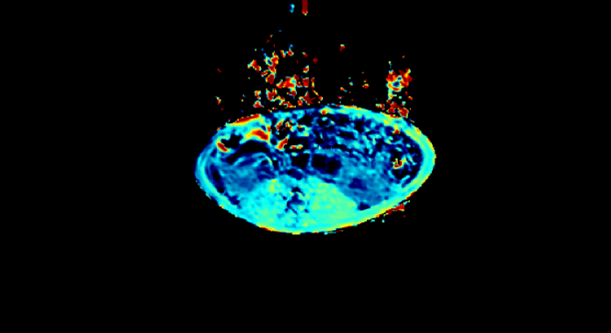

Supplement: Supplementary file 30 — Source data Fig. 5 [file 44321_2024_129_MOESM30_ESM.zip › Source data_Figure5/5C/5C Images/C0 Week3.tif]

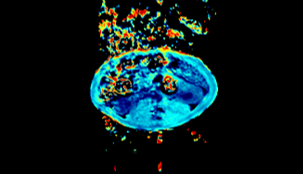

Supplement: Supplementary file 30 — Source data Fig. 5 [file 44321_2024_129_MOESM30_ESM.zip › Source data_Figure5/5C/5C Images/C1 Week2.tif]

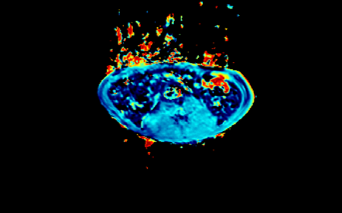

Supplement: Supplementary file 30 — Source data Fig. 5 [file 44321_2024_129_MOESM30_ESM.zip › Source data_Figure5/5C/5C Images/C1 Week3.tif]

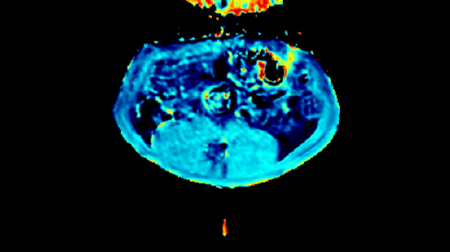

Supplement: Supplementary file 30 — Source data Fig. 5 [file 44321_2024_129_MOESM30_ESM.zip › Source data_Figure5/5C/5C Images/Tx Week2.tif]

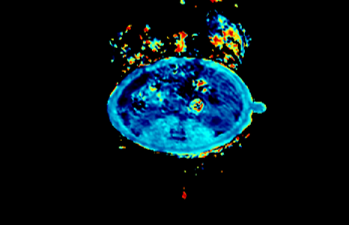

Supplement: Supplementary file 30 — Source data Fig. 5 [file 44321_2024_129_MOESM30_ESM.zip › Source data_Figure5/5C/5C Images/Tx Week3.tif]

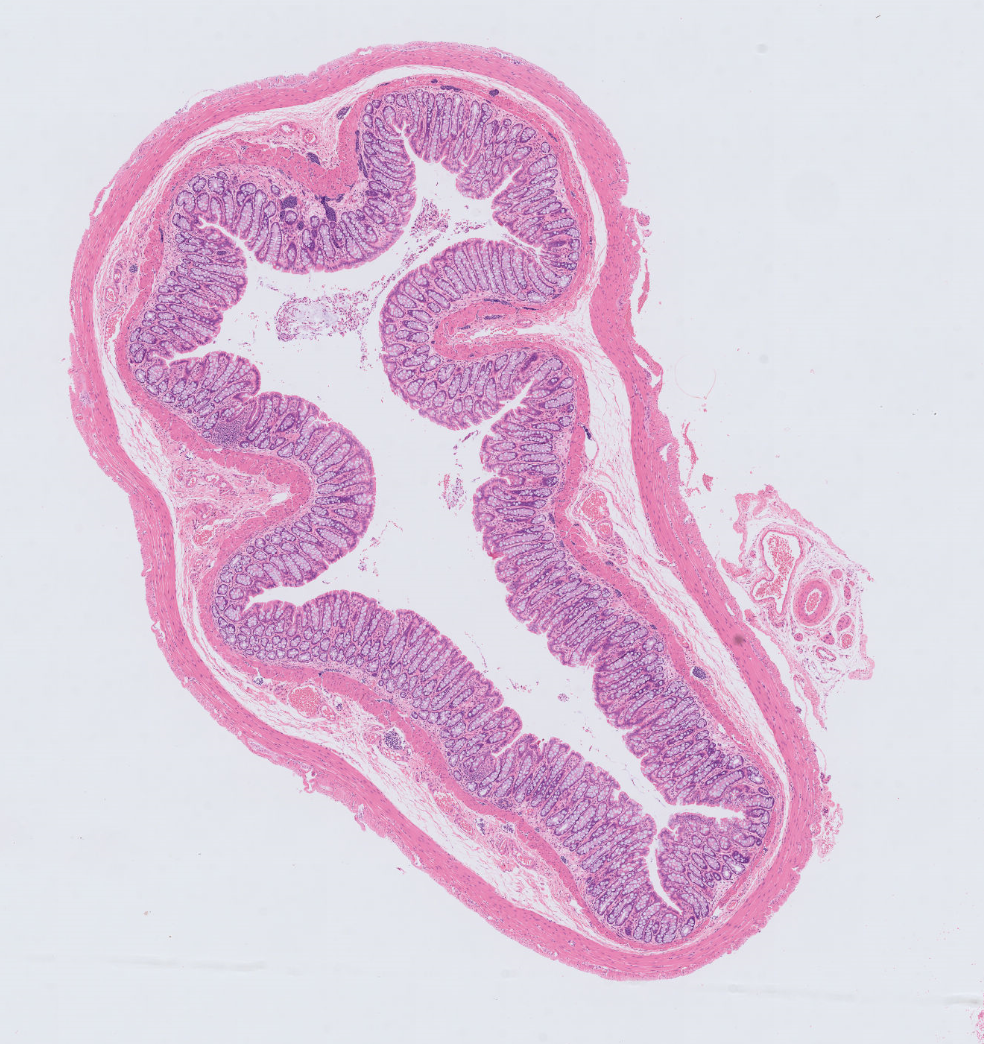

Supplement: Supplementary file 30 — Source data Fig. 5 [file 44321_2024_129_MOESM30_ESM.zip › Source data_Figure5/5D/5D Images/C0+Week2 HE.png]

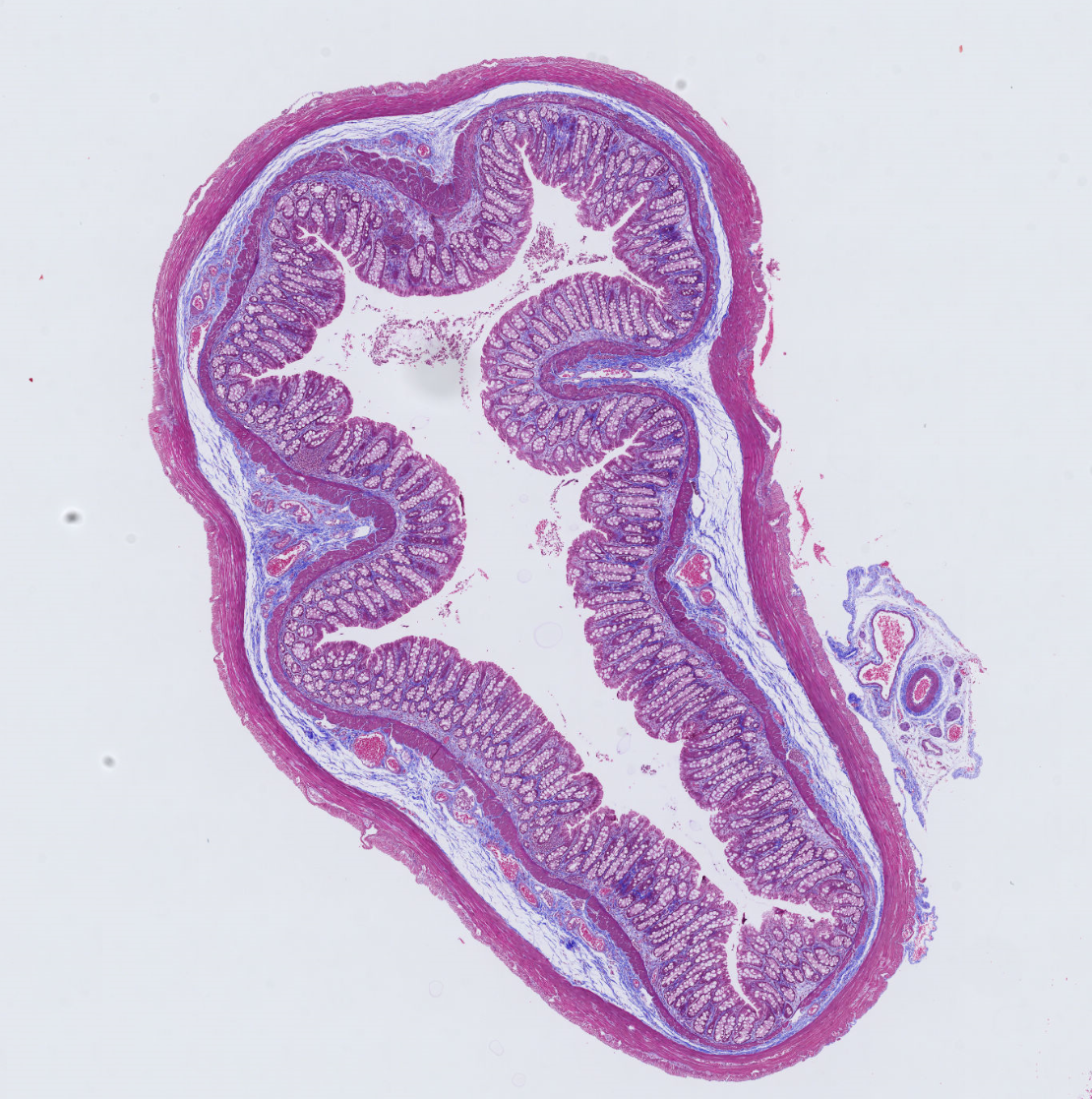

Supplement: Supplementary file 30 — Source data Fig. 5 [file 44321_2024_129_MOESM30_ESM.zip › Source data_Figure5/5D/5D Images/C0+Week2 Masson.png]

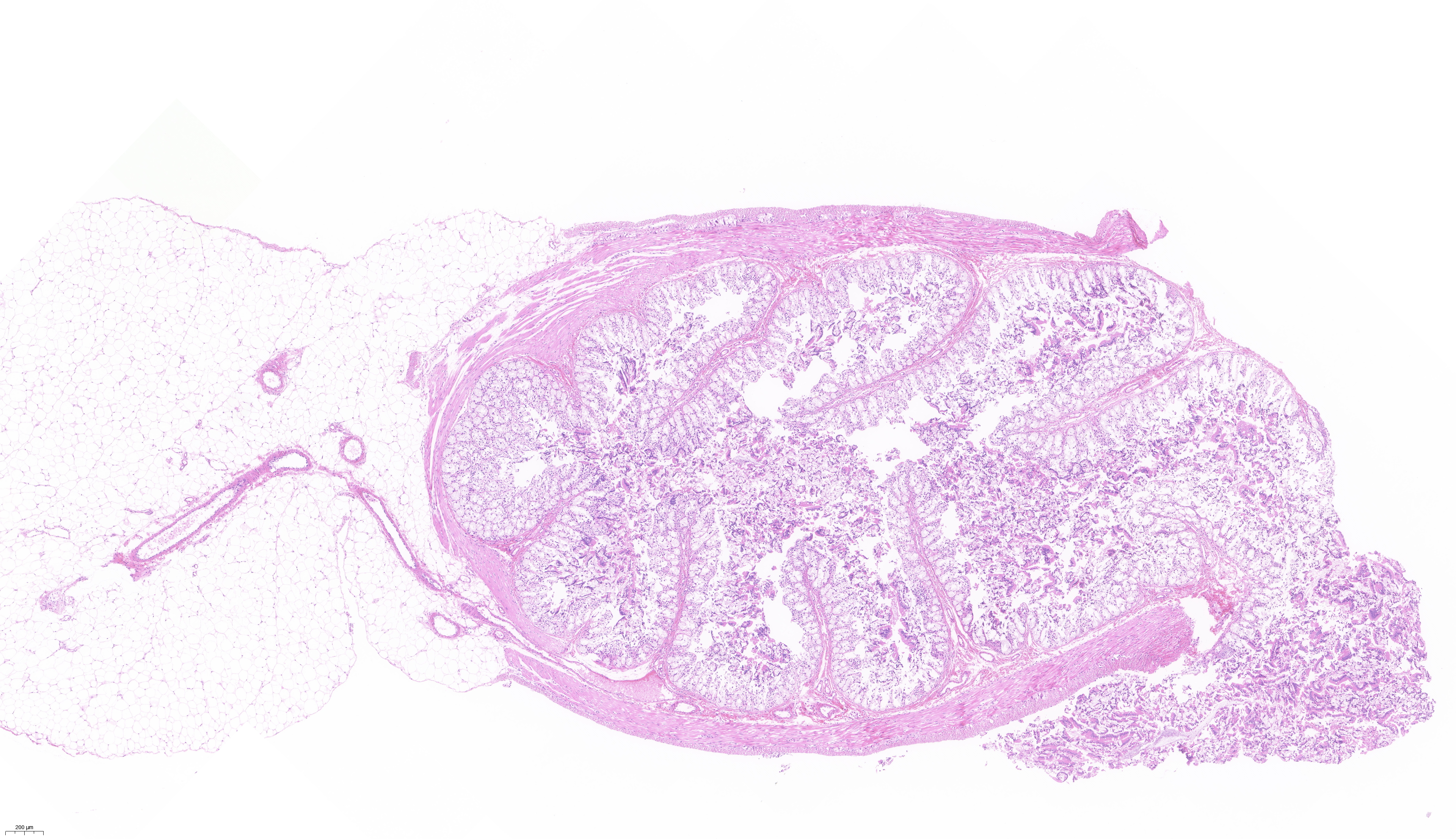

Supplement: Supplementary file 30 — Source data Fig. 5 [file 44321_2024_129_MOESM30_ESM.zip › Source data_Figure5/5D/5D Images/C0+Week3 HE.jpg]

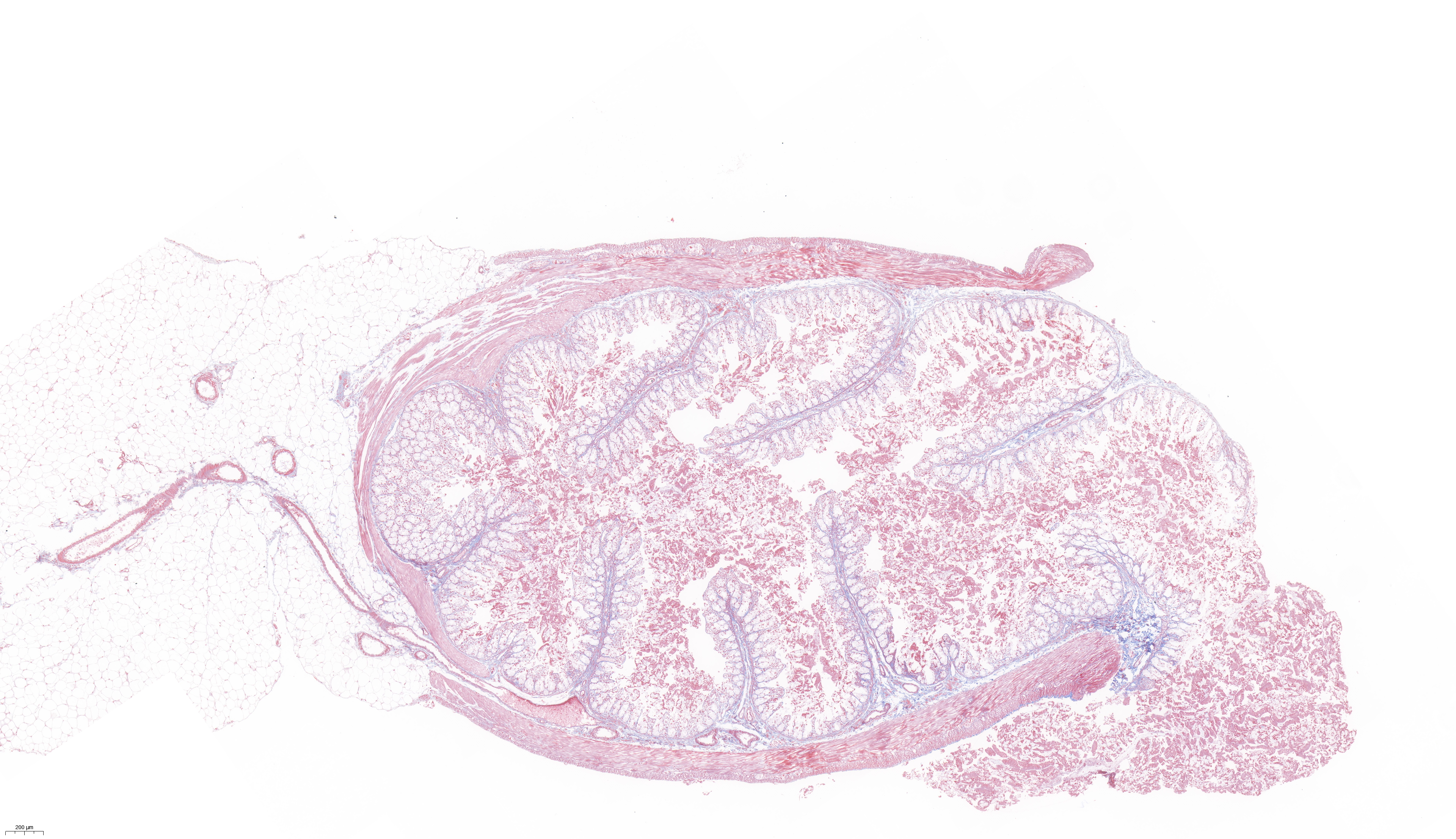

Supplement: Supplementary file 30 — Source data Fig. 5 [file 44321_2024_129_MOESM30_ESM.zip › Source data_Figure5/5D/5D Images/C0+Week3 Masson.jpg]

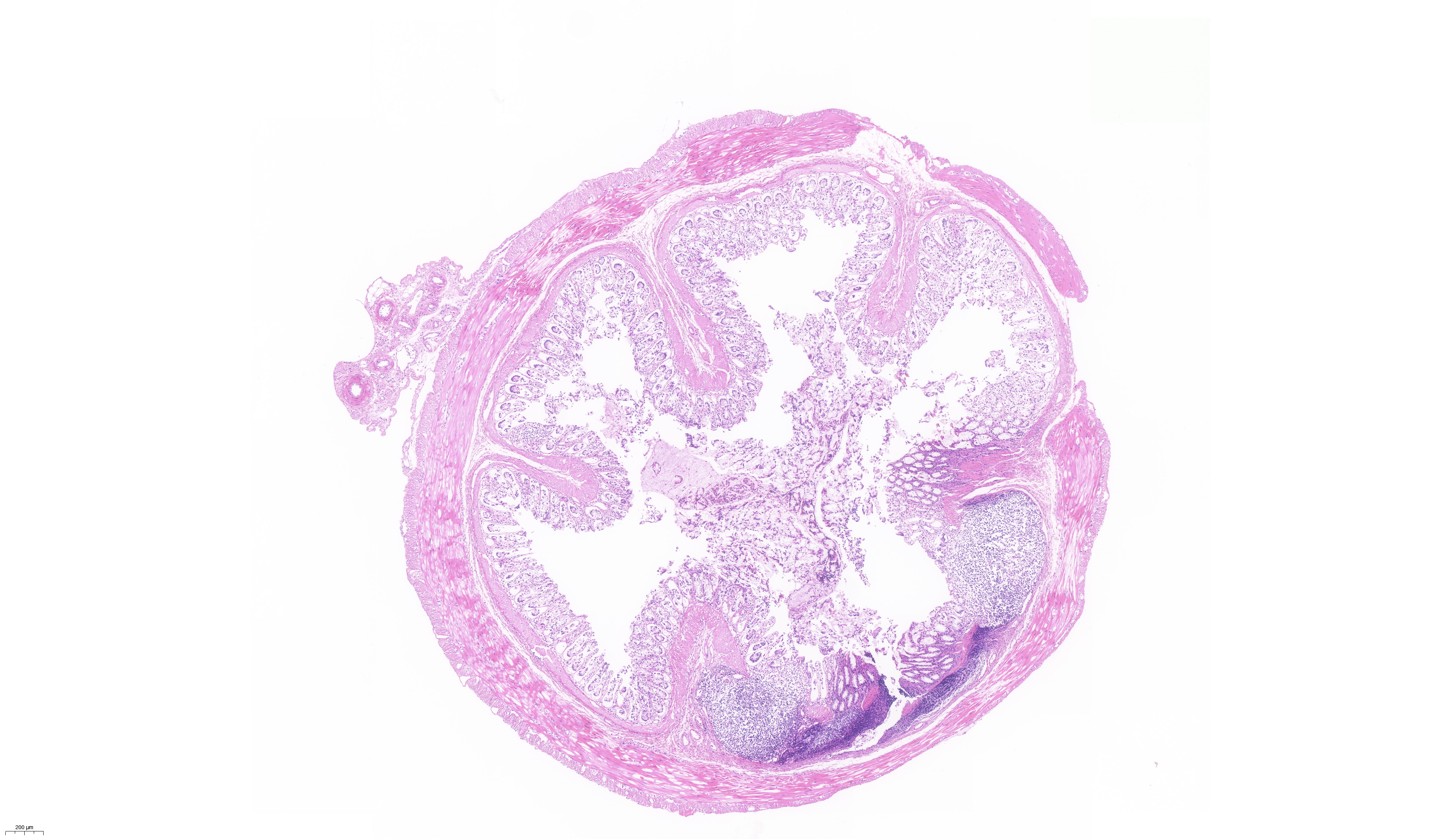

Supplement: Supplementary file 30 — Source data Fig. 5 [file 44321_2024_129_MOESM30_ESM.zip › Source data_Figure5/5D/5D Images/C1+Week2 HE.jpg]

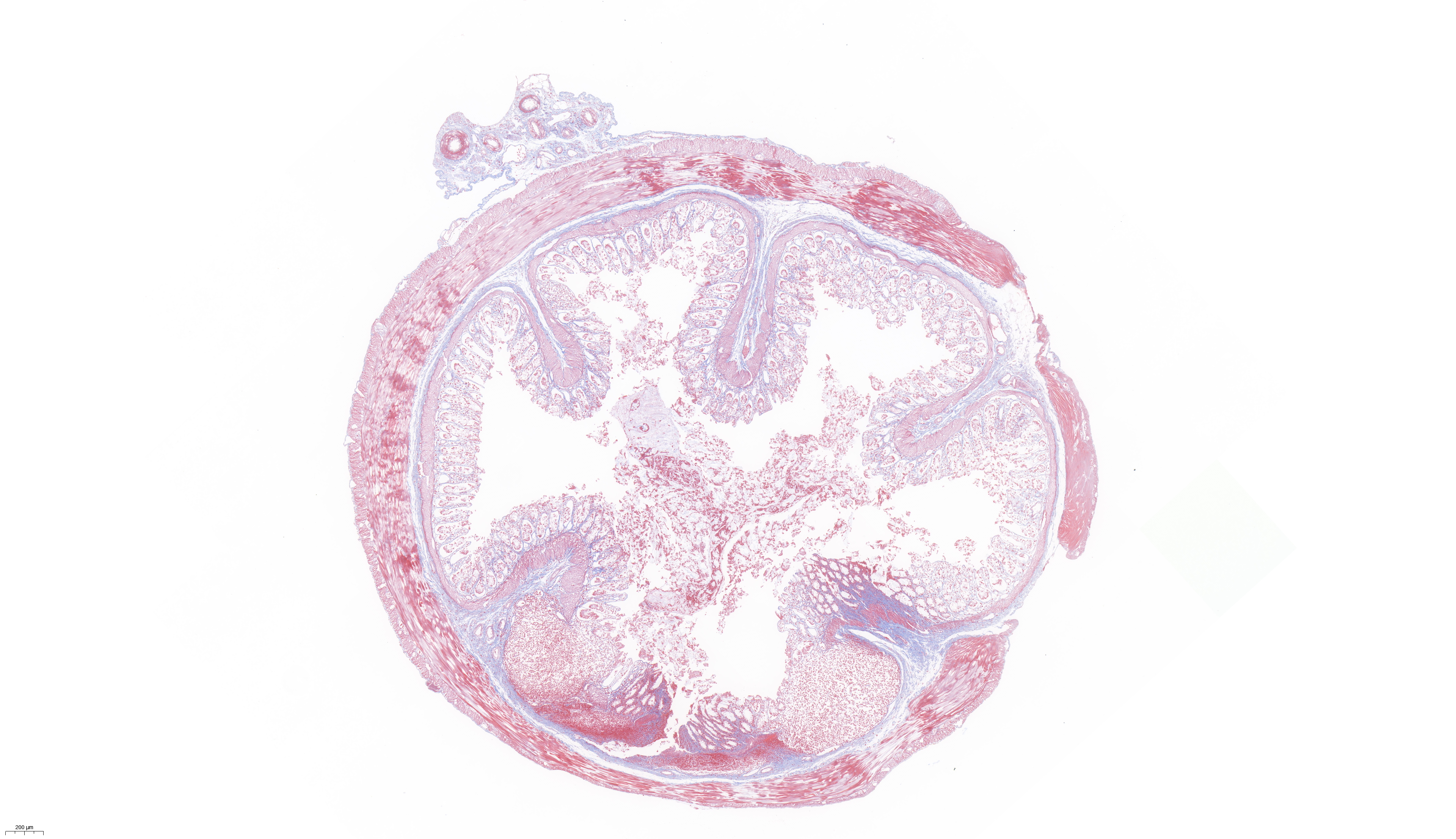

Supplement: Supplementary file 30 — Source data Fig. 5 [file 44321_2024_129_MOESM30_ESM.zip › Source data_Figure5/5D/5D Images/C1+Week2 Masson.jpg]

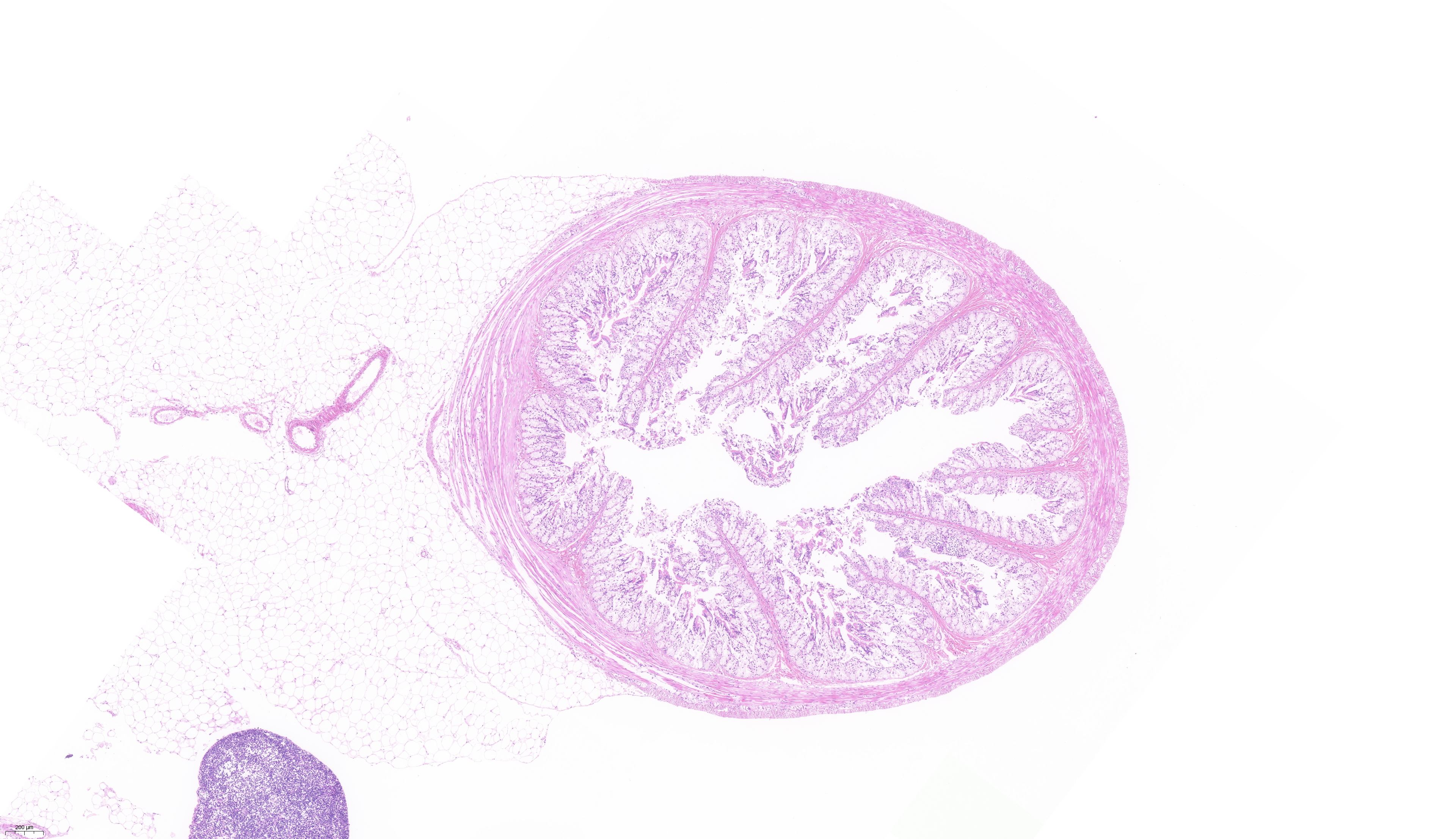

Supplement: Supplementary file 30 — Source data Fig. 5 [file 44321_2024_129_MOESM30_ESM.zip › Source data_Figure5/5D/5D Images/C1+Week3 HE.jpg]

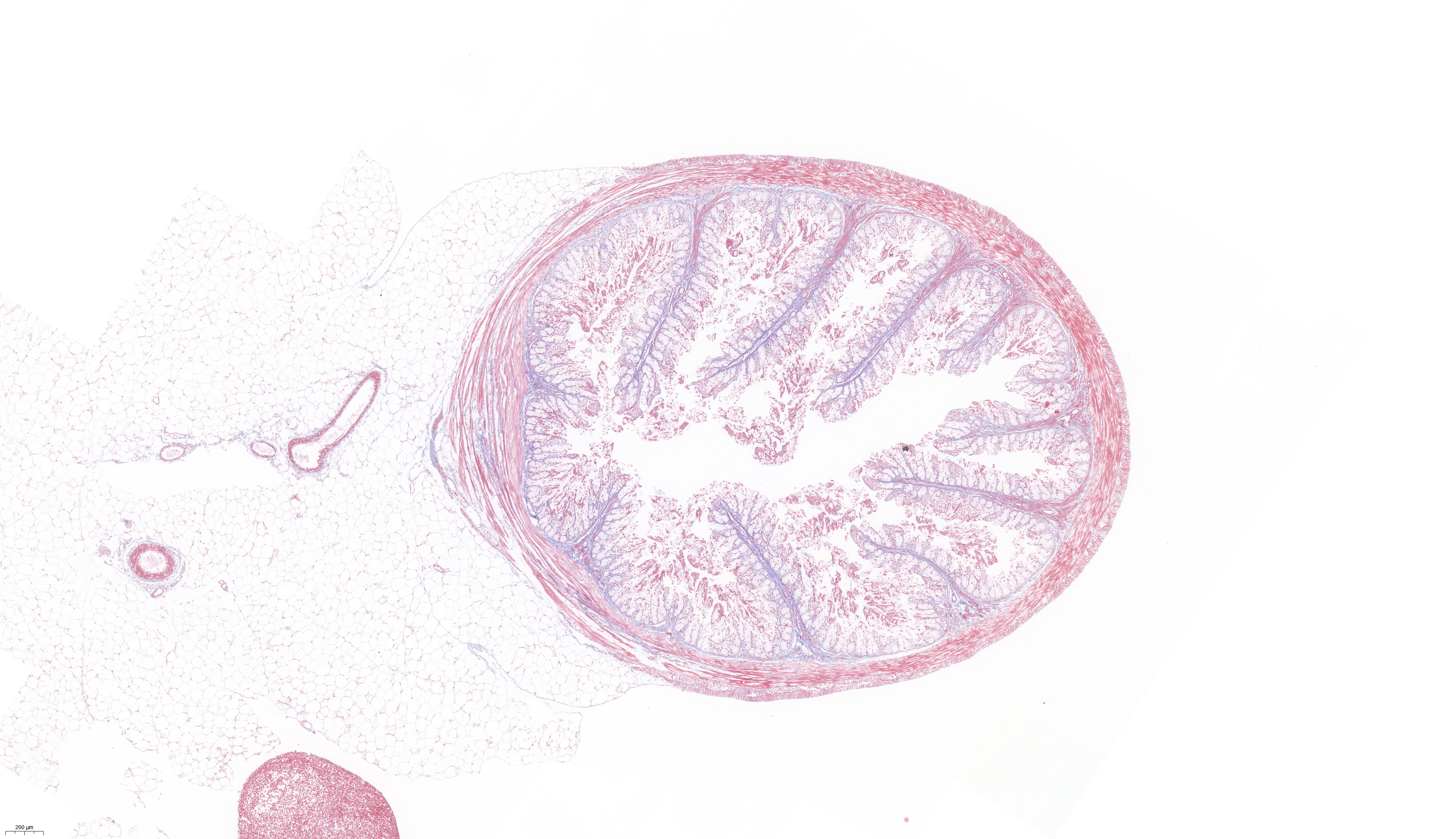

Supplement: Supplementary file 30 — Source data Fig. 5 [file 44321_2024_129_MOESM30_ESM.zip › Source data_Figure5/5D/5D Images/C1+Week3 Masson.jpg]

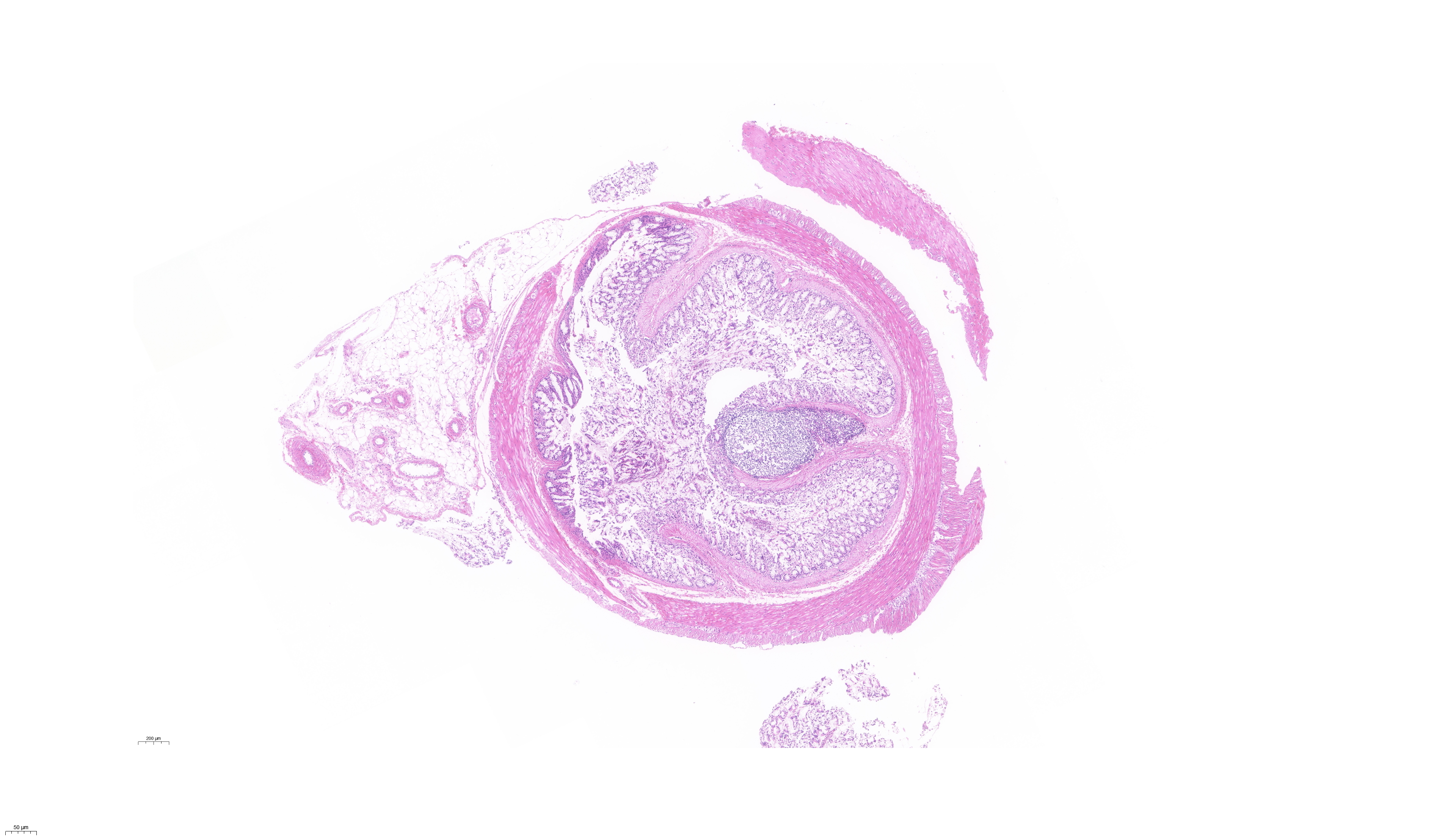

Supplement: Supplementary file 30 — Source data Fig. 5 [file 44321_2024_129_MOESM30_ESM.zip › Source data_Figure5/5D/5D Images/Tx+Week2 HE.jpg]

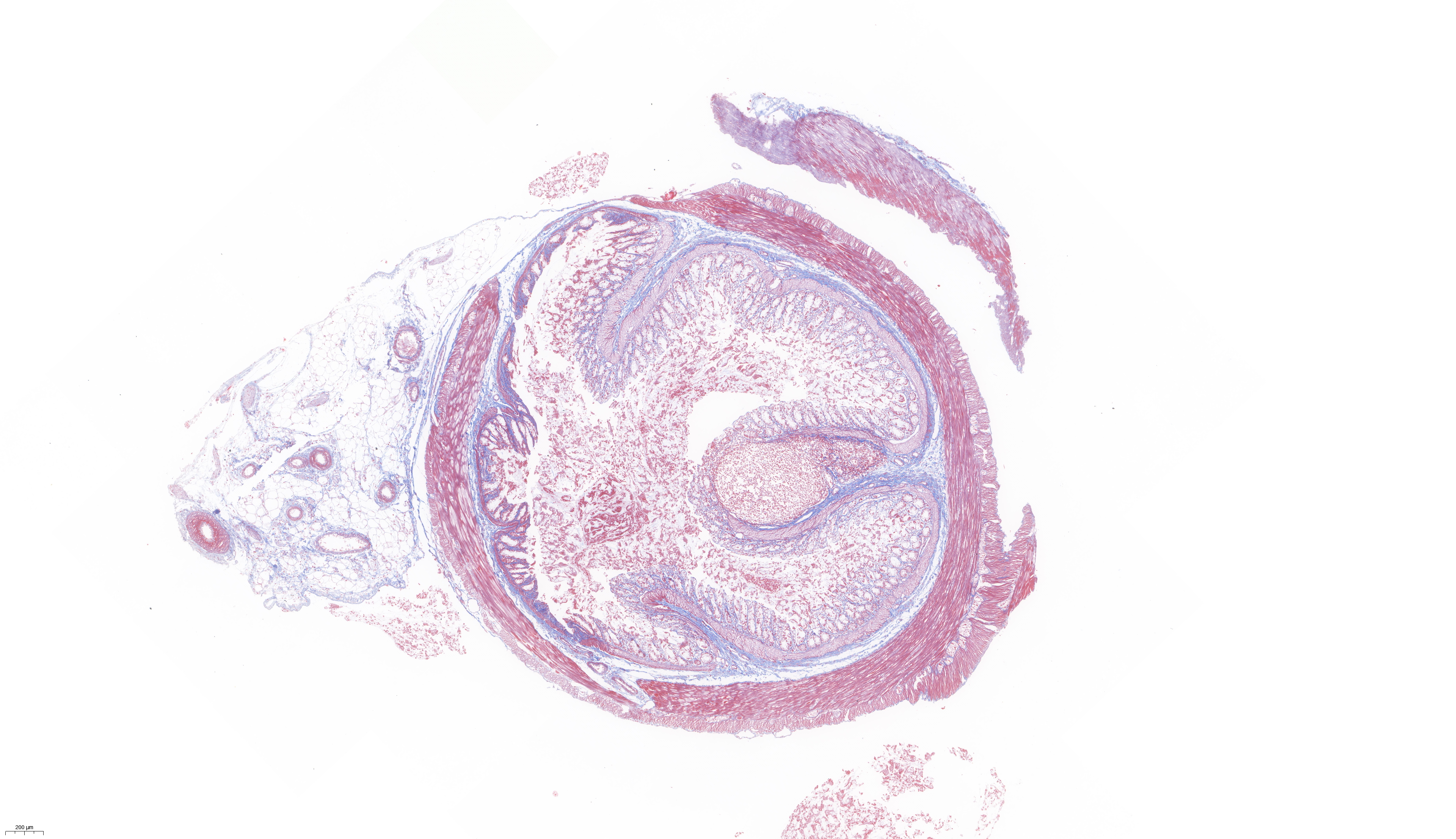

Supplement: Supplementary file 30 — Source data Fig. 5 [file 44321_2024_129_MOESM30_ESM.zip › Source data_Figure5/5D/5D Images/Tx+Week2 Masson.jpg]

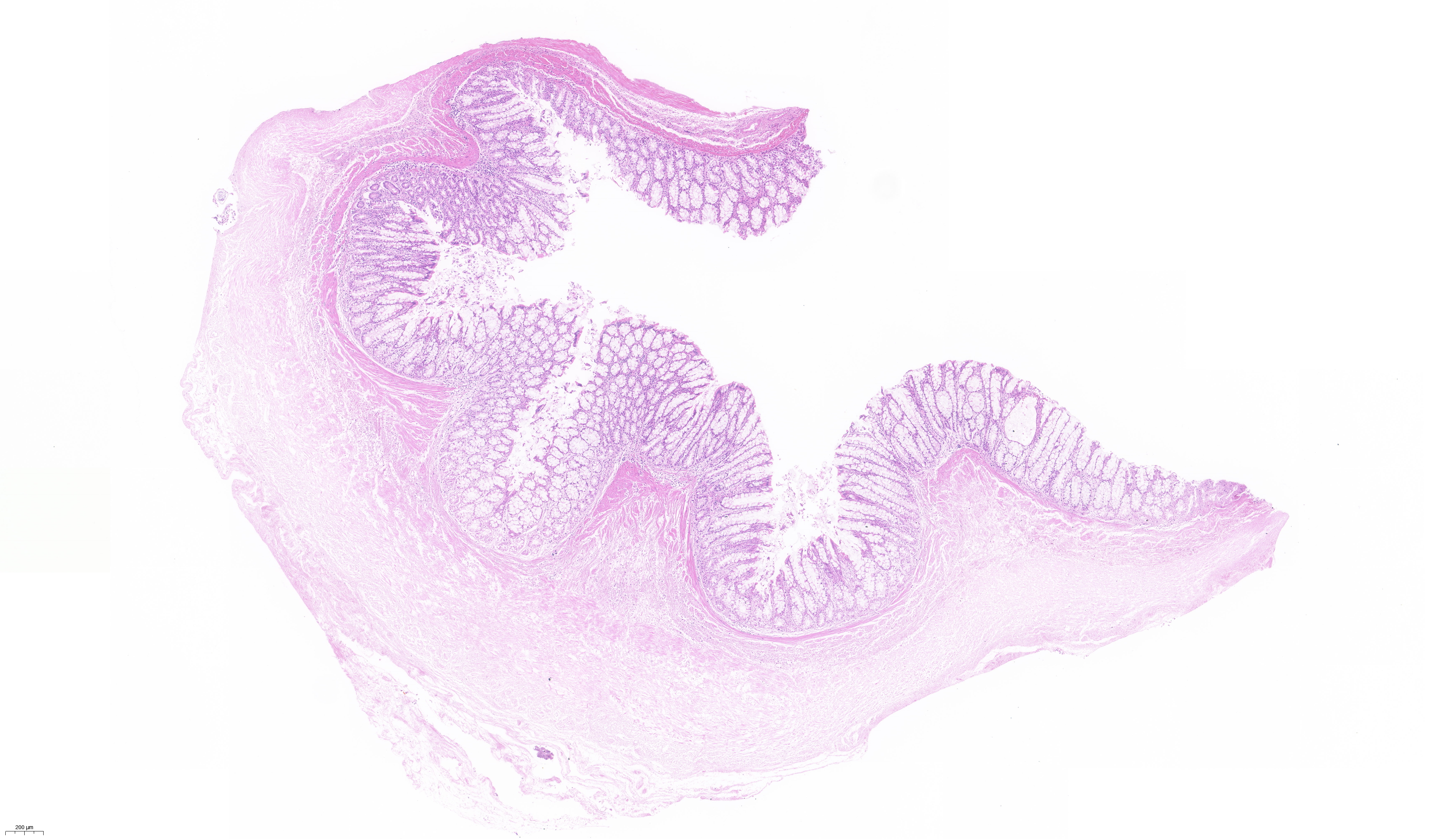

Supplement: Supplementary file 30 — Source data Fig. 5 [file 44321_2024_129_MOESM30_ESM.zip › Source data_Figure5/5D/5D Images/Tx+Week3 HE.jpg]

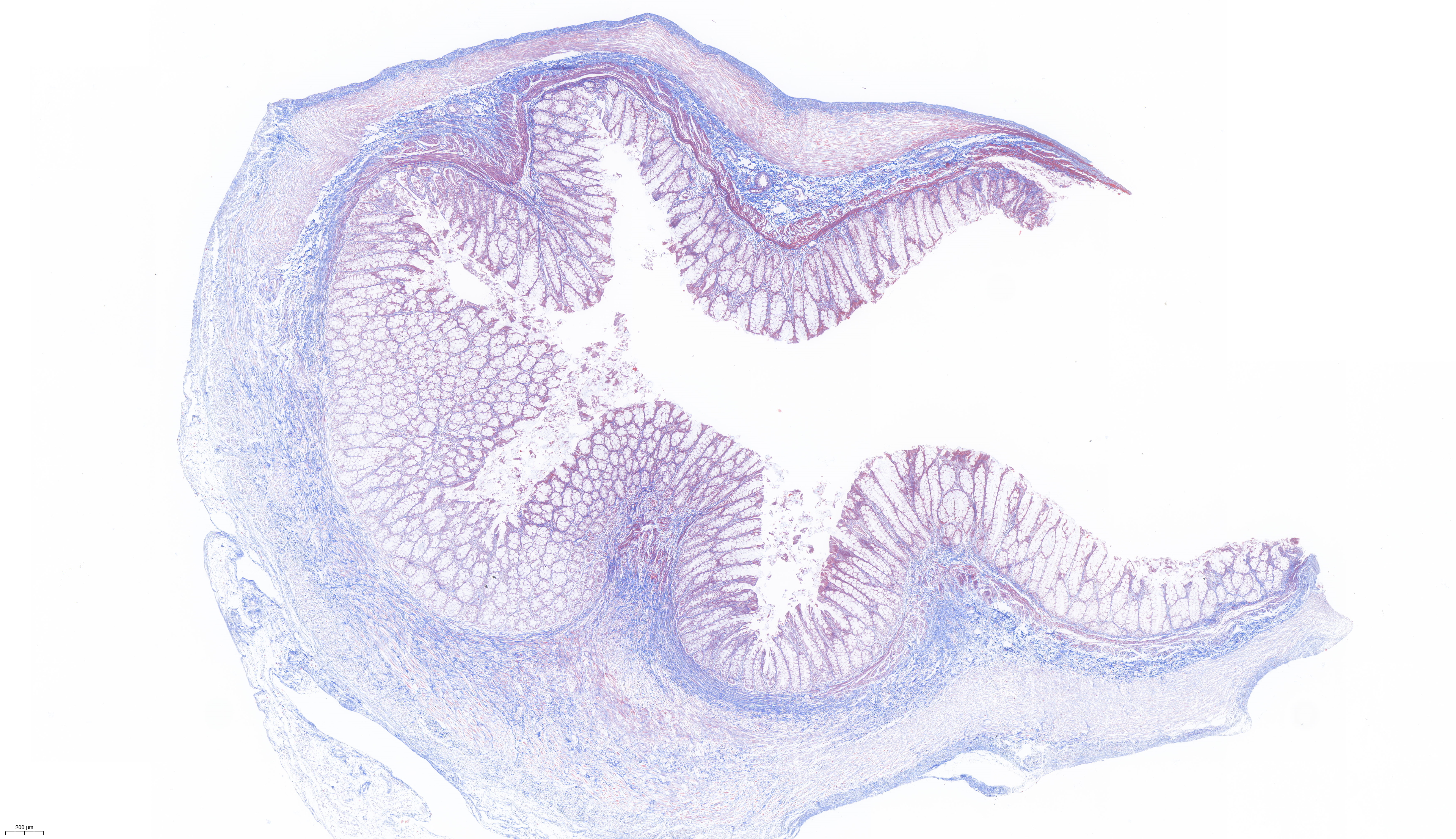

Supplement: Supplementary file 30 — Source data Fig. 5 [file 44321_2024_129_MOESM30_ESM.zip › Source data_Figure5/5D/5D Images/Tx+Week3 Masson.jpg]

**Data file S2. Western blots.**

**Fig. 6d**

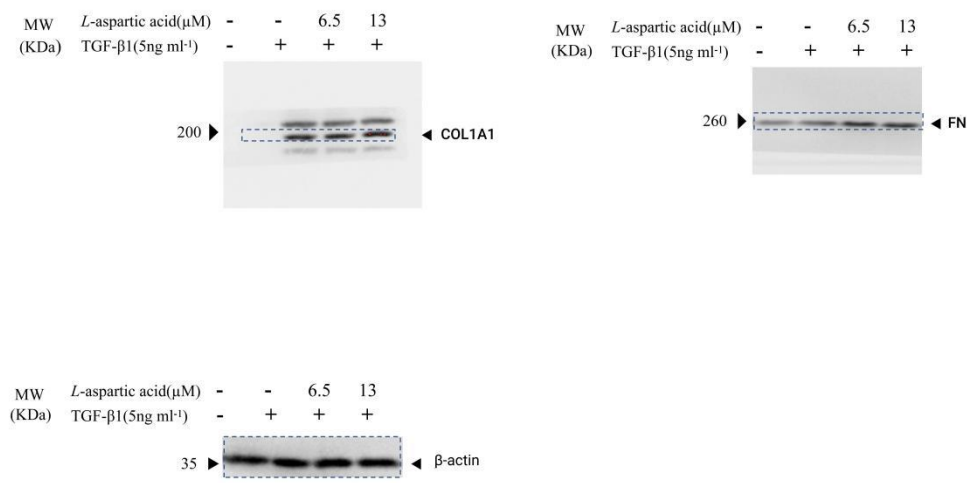

Supplement: Supplementary file 31 — Source data Fig. 6 [file 44321_2024_129_MOESM31_ESM.zip › Source data_Figure6/6D/data_d.pdf]
