# Supplementary material for: Multiomics reveals microbial metabolites as key actors in intestinal fibrosis in Crohn’s disease
Source: EMBO Mol Med. 2024 Sep 13;16(10):11. doi: 10.1038/s44321-024-00129-8 (PMC11473649; doi:10.1038/s44321-024-00129-8)
Supplement: Supplementary file 33 — Expanded View Figures [file 44321_2024_129_MOESM33_ESM.pdf]

Expanded View Figures

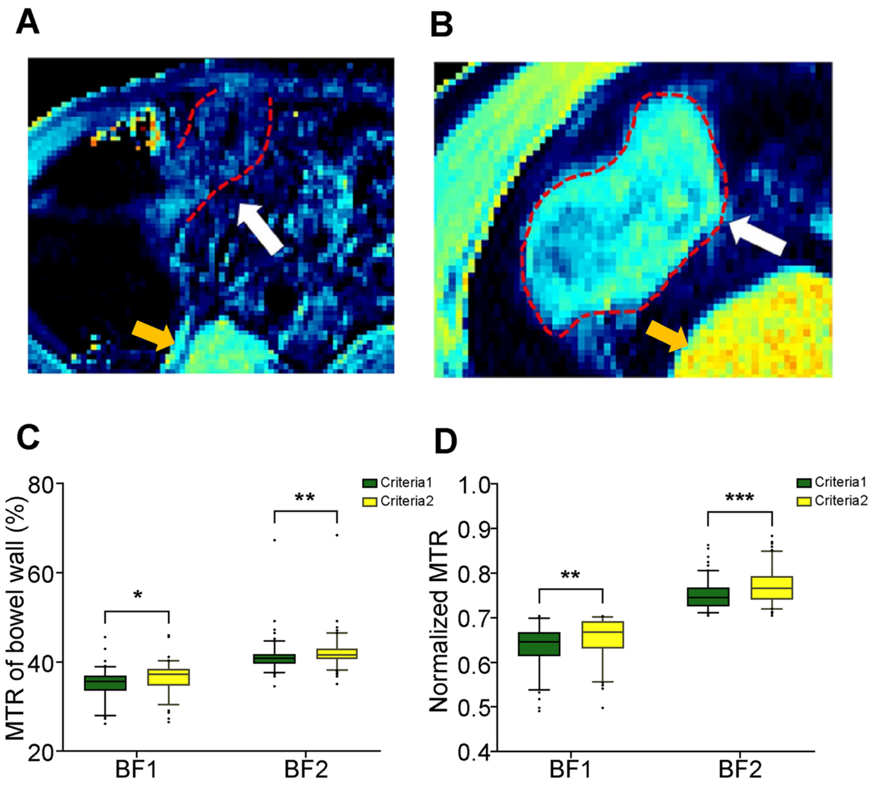

Figure EV1. Assessment of intestinal fibrosis using MTI.

(**A**) Axial color MTR map demonstrating a significant reduction in the MT effect in the terminal ileum in CD (white arrow and red dashed line) compared to that in the right psoas muscle (yellow arrow), indicating none-mild fibrosis (normalized MTR = 0.63). (**B**) Another example demonstrating an MT effect in the terminal ileum in CD (white arrow and red dashed line) that is comparable to that in the right psoas muscle (yellow arrow), indicating moderate-severe fibrosis (normalized MTR = 0.81). (**C**, **D**) Although there are significant differences in the bowel MTR (**C**) and the normalized MTR (**D**) between criterion 1 (normalized MTR from the most severe lesion) and criterion 2 (highest normalized MTR from all lesions) by *T* test in either BF1 ($n = 93$) or BF2 ($n = 121$) patients (all $P < 0.05$), the fibrosis classification of each patient remains consistent regardless of which normalized MTR selection criterion is used. Boxplots (**C**, **D**) represent the interquartile ranges (25th through 75th percentiles, boxes), medians (50th percentiles, bars within the boxes), and the 5th and 95th percentiles (whiskers below and above the boxes). $*P < 0.05$; $**P < 0.01$; $***P < 0.001$. The dots in plots indicate the outliers. Source data are available online for this figure.

© The Author(s)

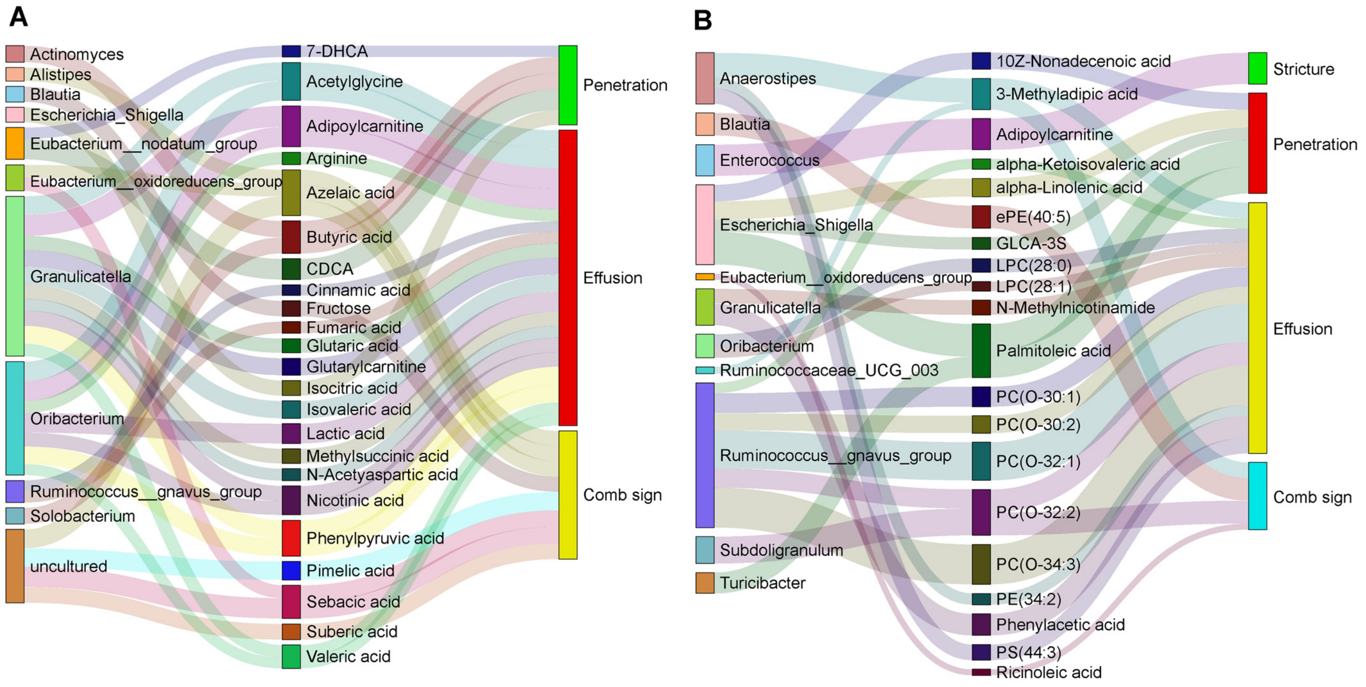

Figure EV2. Putative links between the gut microbiota, metabolites and MRE features in BF1 patients.

(**A**, **B**) Sankey plots indicating bacterial contributors to MRE features mediated by fecal ($n = 23$; **A**) and blood metabolites ($n = 20$; **B**) by mediation analysis. All *P* values of average causal mediation effects <0.05. Color boxes represent the gut microbiota (left column), metabolites (intermediate column) and MRE features (right column), respectively. The line width represents the relative size of *P* value for the correlation between the two "boxes" (wider lines indicate relatively smaller *P* -values). Source data are available online for this figure.

 © The Author(s)

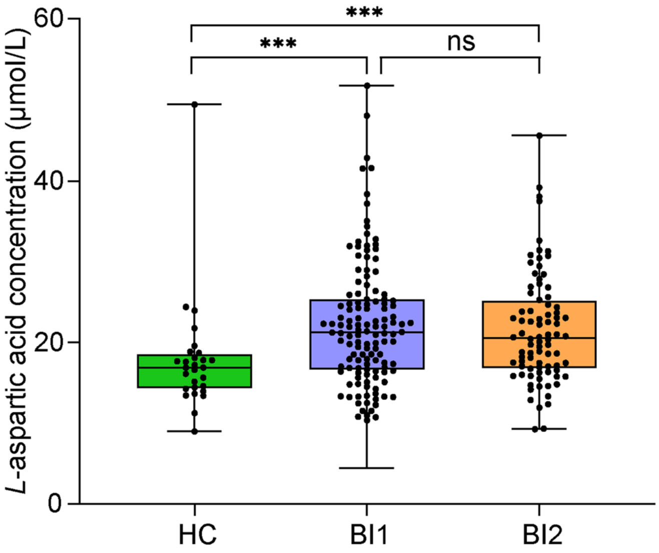

Figure EV3. Comparison of *L*-aspartic acid levels in blood among HCs, CD patients with none-mild inflammation, and CD patients with moderate-severe inflammation.

A total of 130 patients with CD are categorized as having none-mild inflammation (BI1), and the other 84 patients exhibit moderate-sever inflammation (BI2). The boxplots show significant differences in *L*-aspartic acid levels between HCs and BI1 patients ([17.78 ± 7.07] μmol/L vs. [22.12 ± 7.90] μmol/L, $P < 0.001$) as well as between HCs and BI2 patients ([17.78 ± 7.07] μmol/L vs. [21.59 ± 6.80] μmol/L, $P < 0.001$) analyzed by the Mann–Whitney *U* test. However, no significant difference in *L*-aspartic acid levels is observed between BI1 and BI2 patients ([22.12 ± 7.90] μmol/L vs. [21.59 ± 6.80] μmol/L, $P = 0.708$), suggesting that the severity of intestinal inflammation has no significant impact on the *L*-aspartate levels observed in this patient cohort. Boxplots represent the interquartile ranges (25th through 75th percentiles, boxes), medians (50th percentiles, bars within the boxes), and the 5th and 95th percentiles (whiskers below and above the boxes). ***$P < 0.001$; ns, not significant. The dots in the plots indicate individual participants. Source data are available online for this figure.

© The Author(s)
